# Evidence-Based Approaches to Suicide in Older Adults—Zero Suicide

**DOI:** 10.1093/geroni/igaa057.2131

**Published:** 2020-12-16

**Authors:** Luming Li

**Affiliations:** Yale School of Medicine, New Haven, United States

## Abstract

This individual symposium abstract will focus on evidence-based approaches to suicide in older adults, with particular focus on the Zero Suicide Model. Zero Suicide Model is a framework that applies seven essential elements of suicide care (Lead, Train, Identify, Engage, Treat, Transition, Improve). The model provides a systematic approach for quality improvement for suicide prevention and offers implementation strategies for “real-world” clinical settings using the Assess, Intervene, and Monitor for Suicide Prevention (AIM-SP) program for suicide-safer care. The authors will describe implementation of Zero Suicide in general healthcare settings that care for older adults, including health systems and outpatient clinics. The authors will also describe the value of Zero Suicide other settings such as long-term care centers, where older adults are cared for. In addition, the authors will describe future directions for research in the Zero Suicide Model and additional opportunities in public policy for suicide prevention.

